# *MTB*-specific lymphocyte responses are impaired in tuberculosis patients with pulmonary cavities

**DOI:** 10.1186/s40001-016-0242-9

**Published:** 2017-01-26

**Authors:** Jun Wang, Yaping Dai, Jun Liu, Yongmei Yin, Hao Pei

**Affiliations:** 1 Center of Clinical Laboratory, The Fifth People’s Hospital of Wuxi, Affiliated to Jiangnan University, Wuxi, 214005 Jiangsu China; 2 Radiology Department, The Fifth People’s Hospital of Wuxi, Affiliated to Jiangnan University, Wuxi, 214005 Jiangsu China

**Keywords:** Cavity, Tuberculosis, TNF-α, IFN-γ

## Abstract

**Objective:**

Tuberculosis (TB), an infectious disease caused by the bacillus *Mycobacterium tuberculosis (MTB)*, is a global health problem. Because the failing immune response in the lung can lead to formation of a pulmonary cavity, this study was designed to clarify *MTB*-specific lymphocyte responses in TB patients with pulmonary cavities.

**Methods:**

We utilized culture filtrate protein 10 (CFP-10) and early secretory antigenic target 6 (ESAT-6) as immunogenic *MTB* antigens following overnight stimulation of peripheral blood mononuclear cells (PBMCs). By flow cytometry, we then dissected CD4+ and CD8+ T lymphocytes secreting intracellular cytokines of IFN-γ and TNF-α to assess the local immune response of TB patients with pulmonary cavities compared with those having other radiological infiltrates.

**Results:**

As expected, after 16 h of ex vivo activation using both ESAT-6 and CFP-10, the proportions of CD4+IFN-γ, CD4+TNF-α, CD8+TNF-α, and CD8+IFN-γ cells were all markedly increased in 46 patients with TB when compared with 23 household contacts. However, the IFN-γ and TNF-α responses of both CD4+ and CD8+ T lymphocytes were found to be relatively lower in 18 patients who had pulmonary cavities when compared with 28 patients who had radiological infiltrates. Moreover, patients with cavities had higher absolute numbers of neutrophils than patients with infiltrates. Further analysis indicated an inverse correlation between neutrophil counts and the proportions of IFN-γ-secreting T cells.

**Conclusion:**

*MTB*-specific lymphocyte responses are impaired in TB patients with pulmonary cavities that are likely to play an important role in the pathogenesis of cavitary TB.

## Background

Tuberculosis (TB), an infectious disease caused by the bacillus *Mycobacterium tuberculosis (MTB)*, is a global health problem. Overall, a relatively small proportion (5–15%) of the estimated 2–3 billion people infected with *MTB* will develop TB disease during their lifetimes [1]. However, the probability of developing TB is much higher among people infected with human immunodeficiency virus (HIV), indicating that the adaptive immune response plays a vital role in controlling *MTB* pathogenesis. The components of this protective immune response have been investigated with an emphasis on the role of T cells secreting type-1 cytokines, specifically TNF-α and IFN-γ, in the formation of granulomas in the lung [2–6].

However, a failing immune response in the lung may be involved in the formation of pulmonary cavities. With regard to the TNF-α and IFN-γ responses of cavitary TB, several investigators have produced conflicting results. Casarini noted only that IFN-γ but not TNF-α was reduced in cavities compared with areas of infiltrates as measured by ELISA [4], and Condos demonstrated similar results [5]. However, Mazzarella pointed to a reduction in type-1 cytokines from both cavities and areas of infiltrates [6]. Fan et al. also recently demonstrated that the *MTB* antigen-specific Th1 response was decreased when pulmonary TB (PTB) lesions developed in severe cavities [7].

T cells specific for multiple immunogenic antigens of *MTB* have recently been identified, including the Ag85 complex, culture filtrate protein 10 (CFP-10), early secretory antigenic target 6 (ESAT-6), heat shock protein 65, and TB10.4 [8–11]. In particular, ESAT-6 and CFP-10 have been utilized as antigens for the immune diagnosis of *MTB* infection based on the presence of IFN-secreting cells; both have been shown to be more specific and sensitive than tuberculin skin tests [12].

Flow cytometry (FCM) is a powerful technology for characterizing each cell in terms of both the intracellular cytokines, and also activation markers. In addition, FCM is better than enzyme-linked immunosorbent assay (ELISA) to detect cytokines in peripheral blood mononuclear cells (PBMCs) from patients with TB, because ELISA cannot reveal which specific cells are producing and secreting the measured cytokines.

To clarify *MTB*-specific lymphocyte responses in TB patients with pulmonary cavities, we utilized ESAT-6 and CFP-10 as immunogenic antigens and FCM to determine the blood lymphocyte responses of CD4+ and CD8+ T lymphocytes producing and secreting IFN-γ and TNF-α in order to study the local immune responses of subjects with cavitary disease as compared to individuals having pulmonary infiltrates.

## Methods

### Study subjects and ethics statement

Our study included 46 newly diagnosed patients with PTB and 23 household contacts from the physical examination center of our hospital with both TST and T-SPOT negative. All information on the patients was recorded in the clinical database of the Infectious Disease Hospital of Wuxi, China. PBMCs were isolated before treatment from blood samples by means of centrifugation with Ficoll-Hypaque (Sigma). PTB was diagnosed when subjects with clinical and/or imaging features compatible with TB met at least one of the following criteria: positive sputum smear for acid-fast bacilli; positive culture for *MTB*, biopsy suggestive of TB, and/or full response to anti-TB treatment. A radiologist reviewed the posteroanterior chest radiographs (CXRs) of all 48 newly diagnosed PTB patients for the presence or absence of cavities. A respiratory clinician reviewed all CXRs at the same time, with both agreeing on the results.

### Synthetic peptides

Peptides (16- to 18-mers overlapping by 10 aa) corresponding to the sequence of the *MTB* antigens CFP-10 and ESAT-6 were synthesized on an automated peptide synthesizer. For initial screening purposes, peptides were arranged into pools of 9 peptides each in matrix fashion, such that each peptide was uniquely represented in 2 pools [13].

### Intracellular cytokine staining and immunophenotyping

PBMCs were isolated from heparin anticoagulant whole blood and incubated with stimulated antigens using 200 μL of per stimulation in a polypropylene tube. Each patient had four different stimulation setups: negative (medium alone), positive [Phorbol 12-myristate 13-acetate (PMA), each at 5 mg/mL], CFP-10 (10 mg/mL), and ESAT-6 (10 mg/mL). Tubes were vortexed, covered, and kept overnight (16 h) at 37 °C in a 5% CO_2_ incubator. In addition, 10 μg of Brefeldin A (Sigma) was added after 2 h of incubation. Following overnight stimulation, the cells were washed and stained with the following surface antibodies at room temperature for 15 min: energy-coupled dye (ECD)-conjugated anti-CD3 and fluorescein isothiocyanate (FITC)-conjugated anti-CD8. The cells were washed, fixed, and permeabilized (with Caltag reagents A and B) (Caltag Laboratories). The following intracellular antibodies were then added: phycoerythrin–Cy5.5 (PE-Cy5.5)-conjugated anti-IFN-γ and phycoerythrin (PE)-conjugated TNF-α. All antibodies were purchased from Becton–Dickinson.

The cells were incubated for 15 min and then washed and placed on an FC500 (Beckman Coulter) flow cytometer. Negative controls consisting of PBMCs incubated with medium alone were included in each assay. Responses ≥0.03% above the background value were considered to be positive. For phenotypic analysis of CD8 cells, peptide-stimulated cells were gated on CD3+/CD8+ cells and analyzed for expression of TNF-α and IFN-γ. For phenotypic analysis of CD4 cells, peptide-stimulated cells were gated on CD3+/CD8− cells and analyzed for expression of TNF-α and IFN-γ.

### Statistical methods

Statistical analyses were conducted using GraphPad Prism software version 4.0. The unpaired, nonparametric *t* test (Mann–Whitney test), Chi square test, and Spearman rank correlation analysis were performed. Values of *P* < 0.05 were considered to be statistically significant for all analyses.

## Results

### Characteristics of the study population

Our subjects included 46 newly treated TB patients, including 18 patients with cavitary TB, and 28 patients who presented with chest infiltrates on CT scanning. Among the 23 household contacts, there were 11 males and 12 females whose mean age was 50.1 ± 5.6 years. The clinical characteristics of the 46 TB patients and their laboratory data are summarized in Table 1. No significant differences were found regarding age, gender, absolute number of white blood cells (WBCs), monocytes and lymphocytes, or the proportions of CD3+, CD3+/CD4, and CD3+/CD8+ lymphocytes between 18 patients with chest cavities and 28 patients with infiltrates. However, there were significant differences with regard to the absolute number of neutrophils. Patients with cavities had higher absolute neutrophil counts than patients with infiltrates.

**Table 1:** Characteristics of 46 TB patients

Characteristic	TB cases (n = 46)		
	Cavity (n = 18)	Infiltrates (n = 28)	*P* value^a^
Age (years)	55.5	45	0.175
No. % of male subjects^b^	13 (72.2)	18 (64.3)	0.390
Cell count (10^9^/L)^c^			
WBC	6.54 (5.89–8.04)	5.49 (4.76–7.13)	0.105
Monocytes	0.53 (0.37–0.71)	0.39 (0.32–0.61)	0.131
Lymphocytes	1.38 (1.14–1.56)	1.26 (1.02–2.02)	0.831
Neutrophils	4.48 (4.04–5.76)	3.85 (2.56–5.27)	0.019
No. % of positive cells^b^			
CD3+	60.6 (44.3–67.0)	55.6 (52.2–65.4)	0.288
CD3+/CD4+	39.3 (31.1–41.4)	35.4 (29.4–45.5)	0.575
CD3+/CD8+	26.3 (22.5–31.6)	21.9 (16.5–31.8)	0.334

^a^By Mann–Whitney or Pearson’s Chi square test

^b^Data are presented as numbers (%) of individuals except indicated

^c^Data are presented as median numbers (interquartile range)

### Increased percentages of *MTB* antigen-specific cytokine-producing T cells in TB patients

We stained cells for identification by FCM and then tested the proportions of IFN-γ- and TNF-α-producing CD4+ and CD8+ T cells after cells were cultured and stimulated with ESAT-6 and CFP-10, respectively. Results are presented in Table 2. As expected, the percentages of IFN-γ and TNF-α produced by CD4+ and CD8+ T cells after stimulation with ESAT-6 and CFP-10 were significantly higher in TB patients than in household contacts (*P* values all <0.05).

**Table 2:** TB patients have significantly higher proportions of IFN-γ- and TNF-α-producing T cells

	TB (n = 46)	HCs (n = 23)	*P* value^b^
ESAT-6			
CD4			
TNF-α	2.40 (1.90–3.83)	1.71 (1.50–2.40)	0.0003
IFN-γ	0.30 (0.20–0.70)	0.20 (0.13–0.22)	0.0009
CD8			
TNF-α	3.30 (2.20–4.23)	1.71 (1.35–2.50)	<0.0001
IFN-γ	0.65 (0.40–1.75)	0.27 (0.14–0.70)	0.0003
CP-10			
CD4			
TNF-α	2.35 (0.19–0.33)	1.99 (1.63–2.21)	0.0254
IFN-γ	0.40 (0.20–0.63)	0.33 (0.24–0.43)	0.0017
CD8			
TNF-α	2.95 (2.00–4.55)	2.10 (1.60–2.60)	0.0027
IFN-γ	0.60 (0.15–0.52)	0.29 (0.15–0.52)	0.0166

By Mann–Whitney, *NTB* non-tuberculosis, *TB* tuberculosis, *HCs* household contacts

### Impaired *MTB* antigen-specific responses of CD4+ and CD8+ T lymphocytes in PTB patients with cavities

**Fig. 1:**
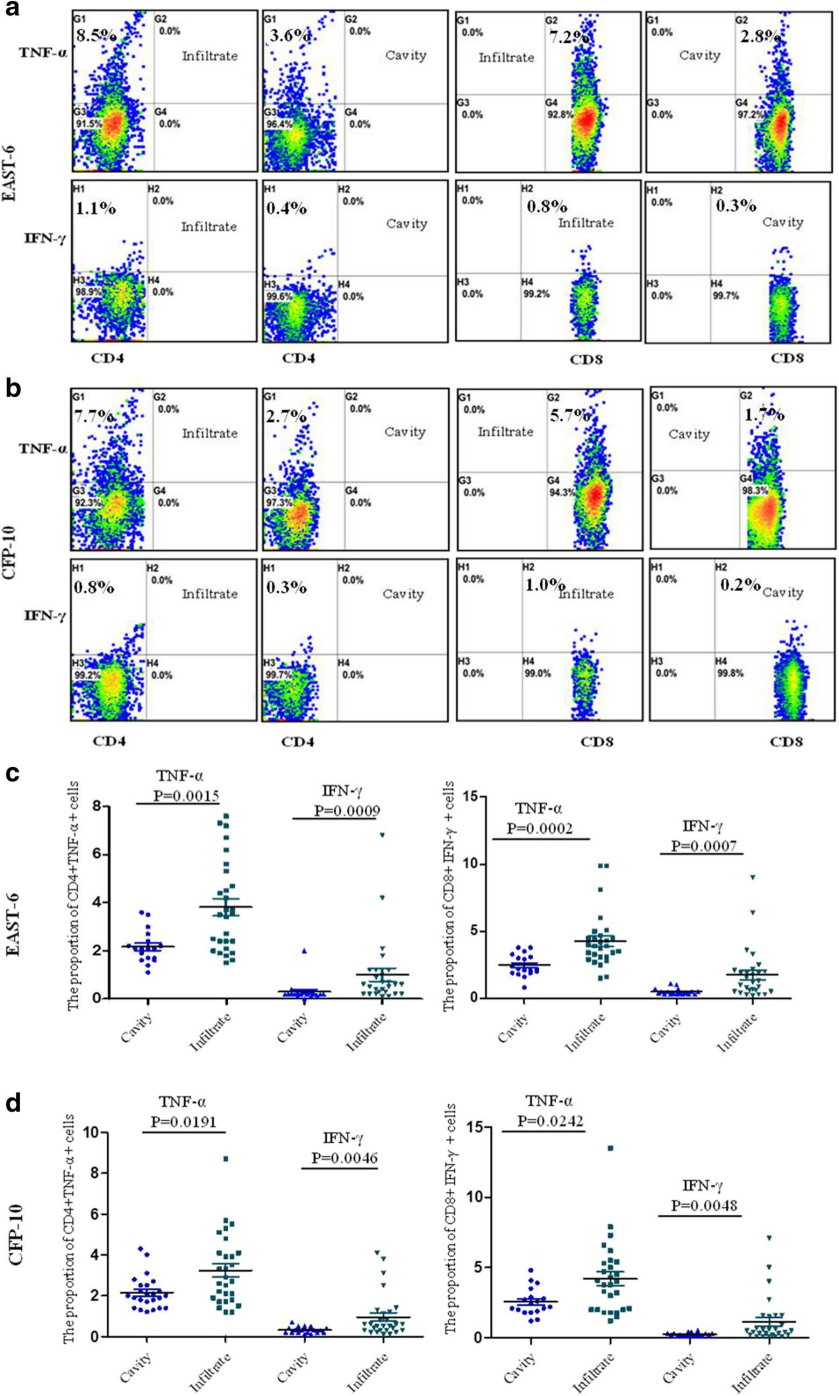
Flow cytometry dot plots of *MTB*-specific CD4+ and CD8+ T-lymphocyte responses in cavitary and infiltrative TB by intracellular TNF-α and IFN-γ staining. **a** Subject with predominant CD4 and CD8 response to EAST-6. **b** Subject with both CD4 and CD8 responses to CFP-10. The percentages in the upper left quadrant denote the percentage of CD4+ TNF-α- or IFN-γ-positive cells; the *right upper* quadrant demonstrates the percentages of CD8 lymphocytes producing TNF-α or IFN-γ cells. Plots of the compared pairs are from the same patient. **c**, **d** Nonparametric t-testing (Mann–Whitney test) was performed and *P* < 0.05 was considered to be a significant statistical difference

*MTB*-specific responses of CD4+ and CD8+ T lymphocytes from TB patients measured by intracellular TNF-α and IFN-γ staining after overnight incubation were shown as FCM dot plots (Fig. 1a, b). TB patients were divided into two groups; on computed tomography (CT) of the chest, 18 patients presented with cavities and the 28 others with infiltrates. The result showed that the TNF-α and IFN-γ produced by CD4+ and CD8+ T lymphocytes in response to ESAT-6 and CFP-10 in the patients with cavities were statistically reduced compared with the radiological infiltrates (*P* values all <0.05) (Fig. 1c, d). The data on the TB patients with cavities and the household contacts were equally low, with *P* > 0.05 (not shown).

### Inverse correlation between the proportions of *MTB*-specific IFN-γ-secreting T cells and neutrophil counts

**Fig. 2:**
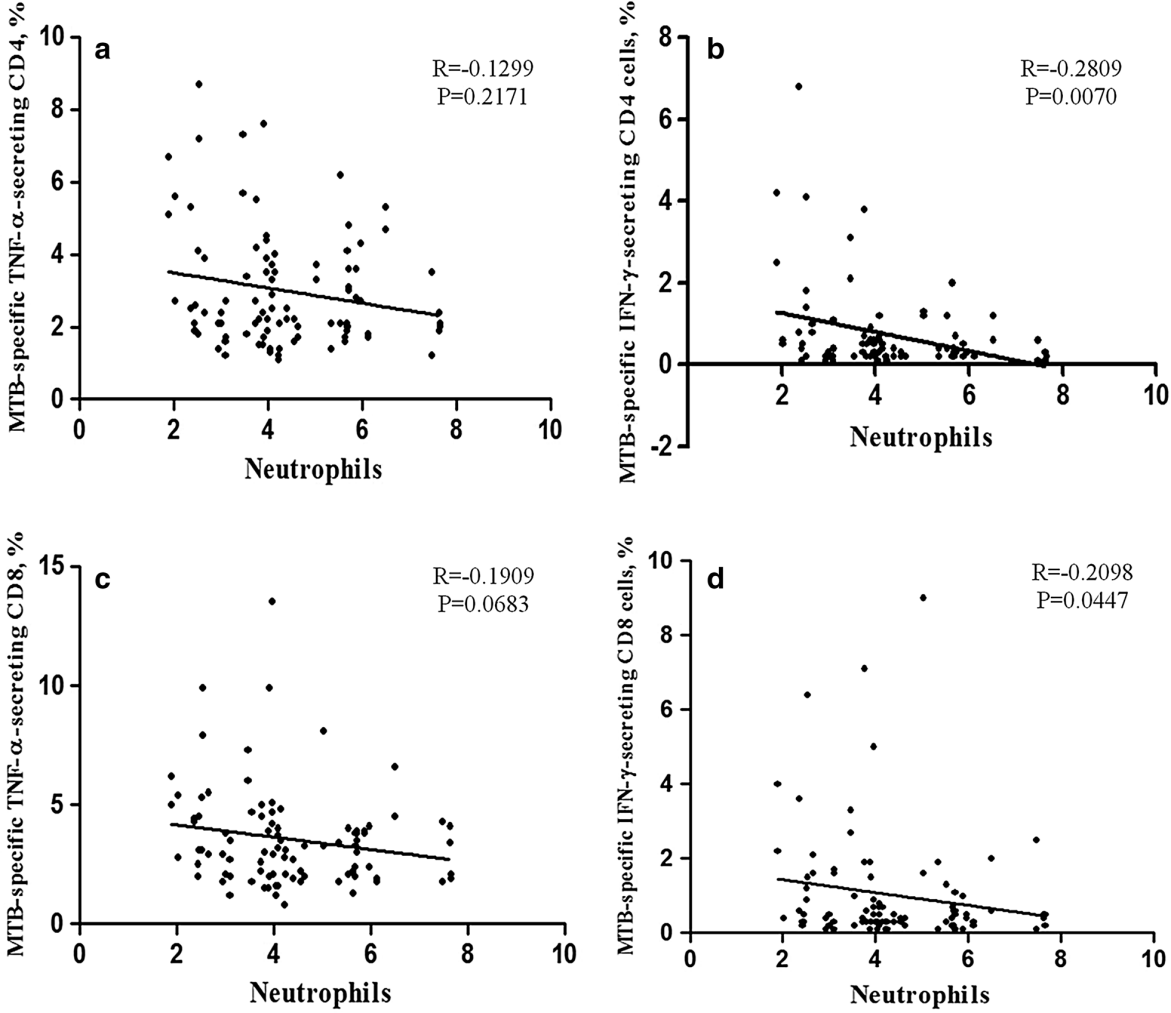
The correlation between the proportion of *MTB*-specific TNF-α- and IFN-γ-secreting T cells (CD4+ and CD8+) and neutrophils counts. A Spearman rank correlation analysis was performed and *P* < 0.05 was considered to be a significant statistical difference

To further determine the impact of high absolute neutrophil counts in patients with cavities on the impaired *MTB* antigen-specific responses, we analyzed the relationship between the proportions of *MTB* antigen-specific IFN-γ- and TNF-α-secreting T cells (CD4+ and CD8+) with neutrophil counts in all TB patients and found no correlation between TNF-α-secreting T cells with neutrophil counts (Fig. 2a, c). However, both IFN-γ-producing CD4+ and CD8+ T cells had significant inverse correlations with neutrophil counts (*R* = −0.2809, *P* = 0.007; *R* = −0.2098, *P* = 0.0447, respectively) (Fig. 2b, d).

## Discussion

Type-1 immune dominance during TB was shown by the predominance of CD4+ and CD8+ T cells responsive to *MTB* antigens by rapid IFN-γ and TNF-α synthesis. These cells were found in both radiologically involved and uninvolved pulmonary sites but were conspicuously reduced in areas of cavity formation [14]. Our results indicate that proportions of TNF-α- and IFN-γ-producing cells within both the CD4+ and CD8+ subsets were significantly reduced in TB patients with cavities compared with those with radiological infiltrates after stimulation of ESAT-6 or CFP-10, respectively, although the activities of IFN-γ and TNF-α were broad and included both beneficial and detrimental effects [15]. IFN-γ and TNF-α may help to recruit cells to the site of infection and promote the antimicrobial activity of macrophages [16, 17]. In addition, TNF-α can lead to TNF-mediated apoptosis of infected macrophages, thus helping to eliminate the pathogen [14]. Therefore, type-1 cytokines, IFN-γ and TNF-α, may generate protective granulomas and enhance the killing of *MTB* within macrophages.

However, the IFN-γ and TNF-α responses of both CD4+ and CD8+ T lymphocytes from TB patients were remarkably impaired in those individuals with pulmonary cavities. The pulmonary cavity has been the classic hallmark of TB and is the site of a very high *MTB* burden. *MTB* infection triggers the recruitment of leukocytes and the activation of intercellular networks, which then results in tissue destruction [18]. Successful immune responses lead to the formation of granulomas and curtailment of the disease process, whereas cavitation indicates a failing immune response [19]. Histological examination also demonstrates a predominance of acid-fast bacilli only at the internal surfaces of the cavities, at which site there are few CD4 and CD8 lymphocytes [19]. The pulmonary cavities are the sites of high *MTB* burdens.

Cavitation has also been reported to be associated with local neutrophilia and relative lymphopenia [14]. Our results show that TB patients with cavities had higher absolute numbers of neutrophils than did patients with infiltrates; moreover, further analysis indicated an inverse correlation between the proportions of *MTB*-specific IFN-γ-secreting T cells and neutrophil counts. The accumulation of neutrophils together with these impaired *MTB*-specific lymphocyte responses probably plays an important role in the pathogenesis of cavitary TB.

Our study also has limitations. The eligible patients represented a fraction of the patients diagnosed with active TB, raising a concern for a selection bias. The antigen of CD4 was endocytosed with the stimulation of PMA and also impaired by the process of permeabilization. Therefore for the phenotypic analysis of CD4 cells, peptide-stimulated cells were gated on CD3+ CD8− cells. Except for these limitations, we conclude that *MTB*-specific lymphocyte responses are impaired in TB patients with pulmonary cavities, which may explain the high *MTB* burden at the cavity site and the severity of cavitary.

## Conclusions

*MTB*-specific lymphocyte responses are impaired in TB patients with pulmonary cavities that are likely to play an important role in the pathogenesis of cavitary TB.

## Abbreviations

TB: tuberculosis
*MTB*: *Mycobacterium tuberculosis*
CFP-10: culture filtrate protein 10
ESAT-6: early secretory antigenic target 6
FCM: flow cytometry
ELISA: enzyme linked immunoabsorbant assays
PBMCs: peripheral blood mononuclear cells
CXRs: chest radiographs
PMA: phorbol myfismte acetate
ECD: energy-coupled dye
FITC: fluorescein isothiocyanate
PE-Cy5.5: phycoerythrin–Cy5.5
PE: phycoerythrin
WBC: white blood cell
IQR: interquartile range
